# The Effects of Trait Emotional Intelligence on Adolescent Substance Use: Findings From a Hungarian Representative Survey

**DOI:** 10.3389/fpsyt.2019.00367

**Published:** 2019-06-07

**Authors:** Bernadette Kun, Róbert Urbán, Borbála Paksi, Mark D. Griffiths, Mara J. Richman, Zsolt Demetrovics

**Affiliations:** ^1^Institute of Psychology, ELTE Eötvös Loránd University, Budapest, Hungary; ^2^Institute of Education, ELTE Eötvös Loránd University, Budapest, Hungary; ^3^Psychology Department, Nottingham Trent University, Nottingham, United Kingdom; ^4^Doctoral School of Psychology, ELTE Eötvös Loránd University, Budapest, Hungary

**Keywords:** emotional intelligence, adolescent substance use, adolescent drug use, adolescent alcohol use, adolescent tobacco use

## Abstract

Previous research has emphasized the importance of emotions in the development of adult and adolescent substance use. There is substantial evidence for deficits in emotional processing among teenagers with substance use, but few studies have investigated the association between emotional intelligence and adolescent substance use. The aim of the present study was to examine the relationship between the use of tobacco, alcohol, and illicit drugs and level of emotional intelligence among adolescents. A representative sample of high school students participated in the study (*N* = 2,380). Substance use patterns were assessed using data from the European School Survey Project on Alcohol and Other Drugs (ESPAD) Survey, and emotional intelligence was assessed with the Bar-On Emotional Quotient Inventory Youth Version. Self-esteem and depressive symptomatology were also assessed to compare their effects on the frequency of substance use with the effect of emotional intelligence. Results demonstrated that greater difficulty in stress management and empathy predicted a higher frequency of tobacco, alcohol, and cannabis use. However, the level of emotional intelligence showed only a weak relationship to substance use habits. Latent profile analyses supported the hypothesis that different emotional patterns and problems underlie different types of psychoactive substances. Using a multiple linear regression model, the present study found that although emotional intelligence is not a key factor underlying substance use habits, it has an individual effect on substance use beyond depressive tendencies and self-esteem. These results can be applied to both drug prevention programs and interventions in substance abuse treatment.

## Introduction

Substance use, especially in adolescence, is a major health issue around the world. It has been well documented that adolescence is a developmental period when teenagers seek new and exciting experiences. It has also been noted that adolescents often experiment with different psychoactive substances such as tobacco, alcohol, and illicit drugs. The Health Behavior in School-Aged Children (HBSC) study is a World Health Organization (WHO) collaborative cross-national study that has been researching the substance use habits of school-aged children since 1983. According to their latest study, 7–56% of 15-year-old students tried smoking cigarettes at the age of 13 years or younger (1). Results indicate that in most countries, regular cigarette smoking and weekly alcohol use starts among those aged 13–15 years. Moreover, it has been shown that in students aged 15 years, 2–32% drink alcohol weekly, and 2–25% of teenagers aged 13 years or younger have had their first experience of being drunk. Lifetime prevalence of cannabis use is between 1% and 29%, and 1–16% of students aged 15 years use cannabis every week (1).

The European School Survey Project on Alcohol and Other Drugs (ESPAD) is another international project, which assesses the substance use habits of adolescents aged 15–16 years every 4 years (2). In reference to the latest survey in 2015, 46% of respondents had smoked cigarettes in their lifetime, 21% were regular cigarette smokers, 81% had drunk alcohol at least once in their lifetime, 13% had been intoxicated during the past 30 days, and 16% had tried cannabis (2). Adolescence also is a very sensitive period because it is typically during this time that indicates which individuals will be experimental, recreational, or compulsive users in adulthood. It has been shown that the earlier the initiation of drug use, the higher the risk for later problematic or dependent drug use (3–6). Moreover, adolescent substance use has several associations with adverse consequences such as increased risky sexual behavior (7), problems with school (8, 9), criminal issues (10), and mental health problems and suicidality (7, 11).

Research has attempted to identify risk and protective factors of adolescent substance use. There are five levels of risk factors: the individual level, the family level, the school level, the community level, and the peer level (12). Among individual risks, several personality factors have been identified including high levels of sensation seeking (13, 14), low levels of impulse control (15, 16), higher levels of neuroticism (17), alexithymia (a personality construct characterized by the subclinical inability to identify and describe emotions) (18), immature self-regulation (19–21), a sense of hopelessness (16, 22), low levels of self-esteem (23, 24), maladaptive coping strategies (25), and low levels of resilience (24, 26). It is clear from these findings that several personality constructs associating with a higher risk of substance use have emotional components. Difficulties in controlling and expressing emotions, having a trait emotional instability, and characterized by low self-esteem increase the risk of substance use problems.

Research examining personality traits, mental health problems, and cognitive processes of emotion regulation have continually emphasized the importance of emotion in adult and adolescent substance use (27). These findings have their roots in earlier clinical observations which were deeply interested in emotional processes of individuals with substance use problems. Psychoanalytic authors have highlighted the presence of undifferentiated, overflowing, dominantly negative and painful feelings, and difficulties in emotional expression in the background of those who abuse psychoactive substances (28–30). Khatzian (28, 31), in his “self-medication theory,” argued that the choice of substance is specific to an individual’s affect-regulation problems, as well as their personality dysfunctions. For instance, individuals characterized by overflowing emotions and immature stress-management skills tend to choose opiates, whereas those described by a tendency to depression, feelings of emptiness, low self-esteem, and difficulties with emotional expression prefer stimulants (31). This theory has not been consistently supported, and the results are mixed (32–34).

Other important evidence of the associations between difficulties in emotional processes and substance use derive from comorbidity studies. Mood disorders and anxiety disorders are the most frequently diagnosed problems among addicted patients (35). It is well-documented that adolescent substance abuse has comorbidity with unipolar depression (11). Depressive mood is a risk factor for adolescents developing severe alcohol and drug-related problems (36). These results support the general notion that individuals frequently use substances to alleviate negative affect.

Currently, emotional regulation has been considered as a multidimensional construct comprising physiological, neural, cognitive, and emotional processes (37). According to this model, adolescents having difficulties in emotion regulation processes (compared to those who do not) are at higher risk for developing substance use disorders (27, 38). Although there is substantial evidence of deficits in emotional processing among teenagers abusing psychoactive substances (39), very few studies have investigated the association between *emotional intelligence* (EI) and adolescent substance use. Furthermore, EI is related to self-regulation, coping, and resilience, which are important individual risk factors of psychoactive substance abuse (40–42).

The concept of emotional intelligence (EI) was first developed by Salovey and Mayer (43), who described the construct as branches of abilities associated with emotions. The four-branch model contains the following: i) perception, appraisal, and expression of emotion; ii) emotional facilitation of thinking; iii) understanding and analyzing emotions; and iv) reflective regulation of emotions to promote emotional and intellectual growth (44). Although this is the most accepted and well-known model of EI, several other theories of EI exist. The other notable concept of EI is the trait EI model which defines EI “as a constellation of emotional self-perceptions located at the lower levels of personality hierarchies” (p. 137) (45). There are several arguments between the theorists of these concepts concerning the components, assessment, and scoring of EI, and both approaches have advantages and disadvantages (46). The distinction between these two EI constructs is now unequivocal, such as the acceptance of so called ‘mixed models’ of EI. Bar-On’s (47) theory represents one of the most well-known ‘mixed models’ of EI, which involve both abilities and personality aspects. Bar-On defined EI as ‘*an array of non-cognitive capabilities, competencies, and skills that influence one’s ability to succeed in coping with environmental demands and pressures*’ (p. 14) (47). In his model, the five domains of emotional-social intelligence are i) the intrapersonal emotional quotient (EQ), ii) the interpersonal EQ, iii) stress management, iv) adaptability, and v) general mood. Each of these components includes a number of closely related competencies, abilities, and facilitators. The intrapersonal EQ scale comprises self-regard, emotional self-awareness, assertiveness, independence, and self-actualization; interpersonal EQ comprises empathy, social responsibility, and interpersonal relationship; stress management comprises stress tolerance and impulse control; adaptability comprises reality-testing, flexibility, and problem-solving; and general mood comprises optimism and happiness (48).

According to the aforementioned studies, the level of EI is expected to be a risk factor in using psychoactive substances. It is presumed that those individuals with good social and emotional skills and competencies can more easily control their substance use habits (21). However, relatively few studies have examined the relationship between psychoactive substance use and EI, and those that have a focus on alcohol use or cigarette smoking [see reviews by Kun and Demetrovics (49), and Resurreccion et al. (50)]. Furthermore, the majority of these studies were conducted on adult populations, and there are only a few studies which have assessed the relationship between EI and any kind of substance use among adolescents (51–55).

Only two studies (51, 55) have examined the relationship between adolescents’ alcohol use and their level of EI, and both of them found a weak negative correlation between alcohol use and EI (r-values were from −0.08 to −0.22). Trinidad and colleagues examined the relationship between cigarette use and level of EI in two surveys published over a number of papers (51–54). According to their results, the lower the level of EI, the quicker the onset of cigarette use and the higher the prevalence of smoking. However, these relationships were also very weak (r-values were from −0.1 to −0.2). At the same time, their results showed that adolescents with more developed EI perceived more negative social consequences of smoking, perceived more success in refusing the offer of cigarettes, and were more reliable in estimating their future smoking behaviors (53). Moreover, EI was reported to be a protective factor regarding acculturation difficulties and perceived social consequences associated with smoking. For those adolescents who were more acculturated in U.S. culture, a higher level of EI could have helped them to perceive the negative social consequences of smoking (54). Vucina and Becirevic (55) also found a very weak relationship between cigarette use and EI.

There is only one study that has examined the association between the level of EI and illicit drug use among adolescents (55). They found very weak negative correlations between level of EI and illicit drug use (*r*-values were from −0.01 to −0.08). Although the participants were asked about their use of several illicit substances (i.e., marijuana, heroin, cocaine, amphetamines etc.), the authors did not compare the usage frequencies of different drugs regarding the level of EI.

Based on this brief overview, it can be concluded that very few studies have examined the associations between EI and adolescent substance use. The existing findings derive from three different samples [the three publications of Trinidad et al. (52–54) reported results from the same sample]. Overall, the association between substance use and the components of EI is significant but weak. However, the studies only used convenience samples, and they did not compare the level of EI among different types of substance users.

Consequently, the aim of the present study was to examine the relationship between adolescent tobacco, alcohol, and illicit drug use and level of EI. First, it was hypothesized that a significant negative correlation would be found between the level of EI and the prevalence of illicit drug use. Second, it was hypothesized that EI would have an individual effect on substance use habits above other significant psychological variables such as depression and self-esteem. Third, according to the self-medication theory (28, 31), it was hypothesized that specific components of EI would relate to different types of substance use. More specifically, deficits in emotional regulation and stress management processes would be risk factors to more frequent use of depressants (alcohol and prescription drugs), and deficits in intrapersonal EI, such as expressing emotions or talking about emotions would be risk factors to more frequent stimulant use (e.g., amphetamine and ecstasy).

## Methods and Materials

### Sample

The target population of the study comprised the entire student population of high schools (grades 9–15) in a Hungarian city (Zalaegerszeg). The target population comprised two subpopulations, grade 9–12 students in general education and grade 13–15 students attending supplementary education. From the first population, 2,245 individuals were randomly selected and stratified by grade and type of school, and the classes in schools were considered as sampling units. In the case of the grade 13–15 population, the entire sample of 824 students, was considered. The total sample population was 3,069 individuals. Overall, 2,492 individuals completed the survey (i.e., 81.2% of the sample, which was considered a good response rate compared to surveys more generally). Of the 2,492 completed surveys, the responses of 2,380 individuals were suitable for analysis. The surveys of students who omitted more than 10% of the questions were excluded from the analysis. The mean age of the participants was 17.0 years (SD = 1.86), with 47.9% being male.

### Measures and Procedure

#### Substance Use

Data on substance use patterns and other related characteristics were obtained from the questionnaires of the ESPAD survey (56, 57), Hungarian version (58). The survey included questions on sociodemographic characteristics as well as lifetime, past year, and past month prevalence of legal and illegal substance use, risks associated with different drug use behaviors, level of perceived availability of different psychoactive drugs, and specific social and health-related problems.

#### Depressive Symptomatology

The ESPAD survey includes the short six-item version of the Center of Epidemiological Studies Depression Scale (CES-D) (59). The scale was developed to screen for signs of depressive tendencies. Participants have to rate how often did they experience the symptoms of depression on a four-point Likert scale (1 = rarely or none of the time; 2 = some or a little of the time; 3 = occasionally or a moderate amount of time; and 4 = most or all of the time). The psychometric properties of the CES-D have been found to be adequate in both adult and adolescent populations (60, 61). This short version of the scale had very good internal consistency in the present sample (Cronbach’s α = 0.81).

#### Self-Esteem

To assess self-esteem, the 10-item Rosenberg Self-Esteem Scale (RSES) was used (62). This instrument was developed to assess self-worth by assessing both positive and negative feelings about the self. Participants have to rate themselves on a four-point Likert scale from *strongly agree* to *strongly disagree*. The unidimensional scale comprises five reversed items (2, 5, 6, 8, 9). The psychometric properties of the RSES have been found to be adequate in both adult and adolescent populations [e.g., Refs. (63, 64)]. The scale had good internal consistency in the present sample (Cronbach’s α = 0.78).

#### Emotional Intelligence

The perceived level of EI was assessed using the Hungarian version of Bar-On Emotional Quotient Inventory Youth Version, Short Form (Bar-On EQ-i YV[S]) (65, 66). This instrument is a self-report measure of EI which assesses personality traits, and social and emotional competencies. Participants have to rate themselves on a four-point Likert-scale by evaluating to what extent they consider the given statement true. The 24 items, in line with the EI model of Bar-On (47), are shared between the following five scales: i) intrapersonal emotional intelligence scale (e.g., ‘*I can talk easily about my feelings*’); ii) interpersonal emotional intelligence scale (e.g., ‘*I care what happens to other people*’); iii) stress management scale (e.g., ‘*I get too upset about things*’); iv) adaptability scale (e.g., ‘*I can come up with good answers to hard questions*’); and v) positive impression scale (e.g., ‘*I do not have bad days*’). The short version does not contain the general mood scale or the inconsistency index. The Hungarian version of the scale includes six reverse items (all of them belonging to the stress management scale). Permission was obtained from the owner of the instrument (Multi-Health Systems Inc) to use the validated Hungarian version of the scale. Three of the scales had good internal consistency (Cronbach’s alpha coefficients: intrapersonal EQ = 0.83, stress management = 0.91, and adaptability = 0.92). The internal consistency of the interpersonal EQ scale (Cronbach’s alpha = 0.71) was acceptable although the positive impression scale’s reliability was arguably questionable (Cronbach’s alpha = 0.60).

#### Procedure

The survey was answered in the classroom in groups of 25–35 students. In each of the classes, a trained interviewer, independent of the school, carried out the data collection. The interviewer informed the participants about the survey and their tasks. Every student received a separate survey that they filled out individually. On all levels of the survey—school, class, and student—data were handled anonymously and presented on a voluntary basis. With the help of the schools, parents were informed about the survey in advance and their consent was obtained. This study was approved by the Scientific Ethical Committee of the research team’s university, and the study was conducted in full compliance with the principles expressed in the Declaration of Helsinki.

### Statistical Analyses

Statistical analyses were conducted with MPLUS 8.1 (67) and Statistical package for the Social Sciences (SPSS) 24.0 programs (68). Latent profile analysis was performed to identify how groups of adolescents differ in their social and emotional competencies. Chi-square tests were applied to determine whether there were differences between the frequencies of substance use (never used versus ever used) in the latent profiles. To examine the possible differences in the prevalence of substance use, a one-way ANCOVA (analysis of covariance) was conducted comparing these latent classes of adolescents, by controlling for the effects of both gender and age. To assess the relationship between substance use habits and EI subscales, correlational analysis was performed using Pearson product-moment correlations. Structural equation modeling (SEM) was used to test the predictive values of social and emotional competencies concerning substance use. Logarithmic transformations were applied to all the outcome variables (which made their distributions more normalized). Gender and age were controlled for in the analyses. During SEM analyses, the multiple linear regression (MLR) estimator was applied for robust to nonnormality. Multiple linear regression was used to test individual effects of EI on substance use, independently from other psychological variables such as self-esteem and depressive symptomatology.

## Results

### Latent Profile Analysis

A *latent profile analysis* was carried out on the 24-item EQ-i YV(S) scale and was suitable for creating groups among adolescents with the emotional and social competencies. Five to eight classes were expected, and therefore, these solutions were tested. The eight-class solution was not acceptable (*p* = 0.6612), so the seven-class solution was verified [AIC (Akaike Information Criteria) = –50,752.466; BIC (Bayesian Information Criteria) = –50,486.919; SSABIC (Sample Size Adjusted Information Criteria) = –50,633.071; entropy = 0.752; L-M-R test (Lo–Mendell–Rubin test) = 70.333; *p* = 0.0412] (**Table 1**).

**Table 1:** Fit indices of the patent profile analysis on Bar-On Emotional Quotient Inventory Youth Version, Short Form (Bar-On EQ-i YV (S)) scale.

Number of latent profiles	AIC	BIC	SSABIC	Entropy	L-M-R test	p
5 profiles	−50,553.52	−50,357.25	−50,465.27	0.73	139.85	**0.013**
6 profiles	−50,692.63	−50,461.72	−50,588.80	0.74	147.93	**0.000**
7 profiles	−50,752.47	−50,486.92	−50,633.07	0.75	70.33	**0.041**
8 profiles	−50,792.16	−50,491.98	−50,657.12	0.75	50.61	0.661

AIC, Akaike Information Criteria; BIC, Bayesian Information Criteria; SSABIC, Sample Size Adjusted Information Criteria; L-M-R test, Lo–Mendell–Rubin test; p, p-value of L-M-R test.

Statistically significant p-values are in **bold**.

All the emotional and social competencies of each group were analyzed. Two groups were found that showed a specific response bias (groups 3 and 5). The members of these groups responded to only 1 and 2 (group 3) or 3 and 4 (group 5) on the Likert scale. For this reason, these two groups were excluded from the further analyses (see **Table 2**).

**Table 2:** Emotional and social competencies of the seven latent profiles, by the factors of the Bar-On EQ-i YV (S).

Profiles	1.	2.	3.	4.	5.	6.	7.	Average of the total sample
N	650	142	40	59	24	241	1219	
Intrapersonal EQ	4.5	4.2	1.2	14.7	16.7	13.5	8.9	7.9
Interpersonal EQ	9.0	11.4	2.4	13.7	16.5	14.3	11.4	11.0
Positive impression	3.9	5.0	1.4	5.0	15.6	9.6	6.6	5.6
Adaptability	6.3	13.5	1.5	14.7	16.6	13.2	9.4	9.3
Stress management	12.1	9.8	16.4	8.3	2.7	11.4	11.0	11.2
Title of the classes	“Calm, under average”	“Adaptive alexithymic”	Biased respondents I. (lower points)	“Impulsive emotionally competent”	Biased respondents II. (higher points)	“Emotional competent”	“Average”	

Average values were synchronized into a 1–18 value scale. This synchronization helps compare the classes better.

Approximately half of the sample was classified into class 7 labeled “*average emotionally intelligent*” adolescents. One-quarter of the sample showed a lower level of EI on all the subscales except stress management. Compared to other classes, they were the best in managing their emotions. Therefore, this (class 1) was labeled as “*calm, under average*” adolescents. Less than 10% of the sample showed an above-average level of EI and this (class 6) was labeled “*emotionally competent*” adolescents. Class 2 were labeled as “*adaptive alexithymic*” adolescents because its members showed high levels of adaptability and interpersonal EI, but they were unable to recognize, express, and manage their emotions effectively. Participants (class 4) who had a high level on all aspects of EI except stress management, were labeled as “*impulsive emotionally competent*” adolescents. Identifying these groups made it possible to execute the comparisons among different types of psychoactive substance use in the next second step of the analysis. The total number of the subsample was 2,311. Their average age was 17.0 years (SD = 1.86), and 47.4% were male.

### Substance Use Habits and Emotional Intelligence

#### Correlation Analysis

According to the outcomes of the Pearson’s correlations, emotional and social competencies show a *weak correlation* with psychoactive substance use (the coefficients did not exceed 0.2 in any of the cases). While a higher level of emotion regulation competence (stress management) was associated with lower level of substance use, higher scores on the adaptability scale (mainly problem-solving) were associated with more frequent licit and illicit drug use (**Table 3**). The interpersonal EQ scale also showed significant negative correlations with the frequency of substance use, but only with tobacco, alcohol, cannabis, inhalants, Gamma Hydroxybutyrate (GHB), and anabolic steroids.

**Table 3:** Correlations between scales of EQ-i YV (S) and lifetime prevalences of different psychoactive drugs.

Psychoactive substance	Interpersonal EQ	Intrapersonal EQ	Positive impression	Stress management	Adaptability
Tobacco smoking use	−0.06**	0.05*	0.01	−0.18**	0.08**
Alcohol	−0.08**	−0.01	−0.00	−0.15**	0.06**
Heavy episodic drinking	−0.13**	0.03	0.04*	−0.14**	**0.04**
Drunkenness	−0.11**	0.06**	0.02	−0.16**	0.06**
Cannabis use	−0.05*	0.04	0.02	−0.08**	0.12**
Inhalants use	−0.04**	−0.02	−0.00	−0.14**	0.08*
Using tranquilizers without a doctor’s prescription	−0.04	−0.02	0.00	−0.18**	0.06**
Ecstasy use	−0.02	−0.02	0.00	−0.07**	0.06**
Amphetamine use	−0.03	−0.02	−0.02	−0.06**	0.05**
Lysergic Acid Diethylamide (LSD) or other hallucinogen use	−0.02	−0.04	0.00	−0.06**	0.10**
Magic mushroom use	−0.04	−0.02	−0.02	−0.05**	0.04*
GHB use	−0.06*	−0.02	0.01	−0.06**	**0.04**
Cocaine use	−0.04	−0.03	0.02	−0.07**	0.06**
Crack use	−0.03	−0.04	0.01	−0.03	0.05*
Heroin use	−0.02	−0.03	0.00	−0.05**	0.06**
Other opiate use	−0.03	−0.01	0.01	−0.01	0.04*
Alcohol use with prescription drug use	−0.02	0.01	0.01	−0.13**	0.10**
Alcohol use with cannabis use	−0.03	0.01	0.00	**−0.03**	0.07**
Sedative use without a doctor’s prescription	−0.07**	−0.03	0.00	−0.06**	0.06**
Anabolic steroid use	−0.04	−0.01	0.02	−0.05*	0.05*
Other drug use	0.02	0.03	0.05*	−0.06**	0.11**

* p < 0.05; ** p < 0.01. Statistically significant p-values are boldfaced.

#### Differences in Substance Use of the Five Latent Profiles

First, analysis tested the possible differences in lifetime prevalence of the different substances (by a dichotomous variable as “never used” and “ever used”) in the latent profiles. According to the chi-square tests, significant differences were only found among those using amphetamines and GHB. The lifetime prevalence of amphetamine and GHB use among the “impulsive emotionally competent” adolescents was significantly higher than among other students (see **Table 4**).

**Table 4:** Chi-square tests on the differences between the lifetime prevalences of amphetamine and GHB use in the five latent profiles.

	Never used *N* (%)	Ever used *N* (%)	*χ*² [degrees of freedom (df)]	*p*
**Amphetamine**				
“Calm, under average”	600 (92.7)	47 (7.3)	11.88 (4)	**0.02**
“Adaptive alexithymic”	136 (95.8)	6 (4.2)		
“Impulsive emotionally competent”	51 (86.4)	8 (13.6)		
“Emotionally competent”	224 (92.9)	17 (7.1)		
“Average”	1,152 (95.1)	59 (4.9)		
**GHB**				
“Calm, under average”	634 (98.0)	13.0 (2.0)	10.01 (4)	**0.04**
“Adaptive alexithymic”	142 (100)	0.0 (0.0)		
“Impulsive emotionally competent”	57 (96.6)	2 (3.4)		
“Emotionally competent”	237 (98.8)	3 (1.3)		
“Average”	1,201 (98.8)	9 (0.7)		

Statistically significant p-values are boldfaced.

To compare the substance use habits of the five latent classes, a one-way ANCOVA was used, controlling for the effects of both gender and age. In **Table 5**, only those results which were significant are presented. Marginally significant differences were found in i) past month alcohol use, and significant differences were found in ii) lifetime use of tranquilizers without a doctor’s prescription [i.e., effect size (ES) ≤ 0.06]. According to *post hoc* analyses, “calm, under average” adolescents consumed more alcohol during the past month than “emotionally competent” adolescents. “Calm, under average” adolescents also used more tranquilizers without a doctor’s prescription in their lifetime than “average” emotionally intelligent adolescents (**Table 5**).

**Table 5:** Results of significant analysis of covariance (ANCOVA) tests comparing substance use prevalence of the five latent profiles.

Psychoactive substance use	Latent profiles	*N*	Mean	SD	df	F	(η²)	*p*
Alcohol use in the past month	Calm, under average	641	4.99	8.38	4	1.99	0.003	**0.09**
	Adaptive alexithymic	141	4.76	7.53				
	Impulsive emotionally competent	57	3.01	3.76				
	Emotionally competent	239	3.44	6.04				
	Average	1,206	4.20	7.26				
Lifetime use of tranquilizers without a doctor’s prescription	Calm, under average	650	0.93	4.83	4	3.30	0.006	**0.01**
	Adaptive alexithymic	142	0.93	4.56				
	Impulsive emotionally competent	58	0.37	1.25				
	Emotionally competent	241	0.65	3.83				
	Average	1,218	0.36	2.22				

Statistically significant p-values are in **bold**.

Ordinary scales were linearized with the midpoints of each category.

#### Structural Equation Modeling

In structural equation modeling (SEM), five outcome variables were chosen which had the highest prevalence among adolescents in this Hungarian city (69): tobacco use, alcohol use, drunkenness, binge drinking, and cannabis use. Three models were tested: in model 1, *lifetime prevalence* of each substance were outcome variables; in model 2, *past year prevalences* were outcome variables; and in model 3, *past month prevalence* was the outcome variable. All the fit indices of the three models were suitable (**Table 6**). In the first model, stress management was a significant negative predictor for the lifetime prevalence of the four variables (i.e., all of them except tobacco use). However, standardized beta values were never higher than 0.25, which showed only weak associations. Lifetime prevalence of drunkenness was predicted by stress management at the highest level (β = –0.25; *p* < 0.001), which meant that the lower the level of stress management skills, the higher the prevalence of drunkenness in their lifetime (**Table 6**).

**Table 6:** Standardized β coefficients and R^2^ of the three models on EQ-i YV (S) scales and psychoactive substance use prevalences.

Outcome variables	Standardized β coefficients					R^2 $^
	**Intrapersonal EQ**	**Interpersonal EQ**	**Stress management**	**Adaptability**	**Positive impression**	
**Model 1. Lifetime prevalences**						
Drinking alcohol #	0.03	−0.04	−0.20***	0.05	−0.07	5%
Drunkenness #	0.13***	−0.10**	−0.25***	−0.00	−0.06	9%
Binge drinking #	0.06*	−0.17***	−0.18***	0.04	0.05	7%
Cannabis use #	0.01	−0.16***	−0.13***	0.19***	−0.02	6%
**Model 2. Past year prevalences**						
Drinking alcohol #	0.05	−0.14***	−0.19***	0.07*	−0.01	6%
Drunkenness #	0.11***	−0.16***	−0.22***	0.02	−0.01	9%
Cannabis use #	−0.01	−0.16***	−0.11***	0.15***	0.03	4%
**Model 3. Past month prevalences**						
Drinking alcohol #	0.07*	−0.17***	−0.17***	0.04	0.08*	7%
Drunkenness #	0.06*	−0.21***	−0.19***	0.04	0.08**	8%
Cannabis use #	−0.02	−0.18***	−0.08***	0.15***	0.07*	3%
Daily smoking Ð	0.04	−0.12**	−0.17***	0.04	0.02	5%

Statistically significant correlations are boldfaced.

#, logarithmic transformation was performed, and gender and age were controlled; $, effects of gender and age were excluded; Ð, probit coefficient.

*p < 0.05; **p < 0.01; ***p < 0.001. Fit indices of the models: Model 1: χ^2^ = 3,328, df = 366, Root Mean Square of Approximation (RMSEA) = 0.058 90% CI [0.057–0.060], Comparative Fit Index (CFI) = 0.918, Tucker-Lewis Index (TLI) = 0.902; Model 2: χ^2^ = 3,283.4, df = 347, RMSEA = 0.06 [0.058–0.062], CI = 0.917, TLI = 0.904; Model 3: χ^2^ = 3,316.4, df = 366, RMSEA = 0.058 [0.056–0.060], CFI = 0.917, TLI = 0.901.

The other notable factor was the interpersonal EQ scale, which significantly predicted all the substance use outcome variables except the lifetime prevalence of alcohol use. As **Table 6** shows, the lower the level of interpersonal EQ skills, the higher the lifetime prevalence of drunkenness, binge drinking, and cannabis use. However, the intrapersonal EQ scale had a significant positive relationship with the lifetime prevalence of drunkenness and binge drinking, although the beta coefficients were very low. Adaptability only predicted lifetime prevalence of cannabis use positively. These social and emotional competencies only explained 5–9% of the variance of lifetime substance use.

Past year prevalences were predicted the most by stress management and interpersonal EQ scales. The outcomes were the same as above (i.e., adolescents who used more alcohol and cannabis during the previous year had a lower level of these emotional competencies than others). Intrapersonal EQ was also a positive predictor of past year prevalence of drunkenness. Adaptability showed a similar relationship with past year prevalence of cannabis use. These beta coefficients were also very low, and EQ-i scales explained only 4–9% of the variance of past year substance use. Finally, the results were the same regarding past month prevalences of substance use but the Positive Impression Scale was also a significant predictor of drinking, drunkenness, and cannabis use. Daily smoking was only predicted by stress management and interpersonal EQ. All of the EQ-i scales explained 3–8% of the variance of current substance use (**Table 6**).

#### Effects of Emotional Intelligence on Substance Use According to Other Psychological Variables

Multiple linear regression applied the five EQ YV(S) scales and the total scores on the self-esteem and depressive symptomatology scales as independent variables. Eight analyses were run on the following dependent variables: i) lifetime prevalence of tobacco smoking, ii) past month prevalence of tobacco smoking, iii) lifetime prevalence of alcohol use, iv) past month prevalence of alcohol use, v) lifetime prevalence of drunkenness, vi) past month prevalence of drunkenness, vii) lifetime prevalence of cannabis use, and viii) past month prevalence of cannabis use. Linear transformations were used for all of them.

The analysis showed that self-esteem and depressive symptomatology did not explain more of the variance of substance use habits than EI (**Table 7**). Findings showed that only between 2% and 6% of the variance was explained by EI, self-esteem, and depressive symptomatology combined. According to the values of the standardized beta coefficients, it appears specific EI components have independent explanatory power over substance use compared to self-esteem and depressive symptomatology. Although all of the substance use habits were predicted by EI, the level of self-esteem predicted only lifetime prevalence of alcohol use and drunkenness and past month prevalence of alcohol use. At the same time, depressive tendencies predicted only past month prevalence of alcohol use. In every case, standardized beta coefficients of self-esteem and depressive symptomatology were lower than the beta coefficients of EI. In sum, although EI explained only a small amount of the variance of substance use habits, this relationship exists independently from self-esteem and depressive symptomatology.

**Table 7:** Multiple regression models testing the effects of emotional intelligence, self-esteem, and depressive symptomatology on substance use habits.

Outcome variables	Standardized β coefficients					Self-esteem	Depressive symptoms	R^2^
	**EQ YV(S) scales**							
	**Intrapersonal EQ**	**Interpersonal EQ**	**Stress management**	**Adaptability**	**Positive impression**			
**Lifetime prevalence**								
Tobacco smoking	0.036	−0.131***	−0.182***	0.092***	−0.010	−0.007	0.014	**5%**
Alcohol use	−0.026	−0.120***	−0.175***	0.066	−0.013	0.082*	0.010	**4%**
Drunkenness	0.052*	−0.171***	−0.188***	0.054*	0.005	0.071**	0.001	**6%**
Cannabis use	0.018	−0.117***	−0.0.46	0.143***	0.002	−0.010	0.032	**3%**
**Past month prevalence**								
Tobacco smoking	0.016	−0.113***	−0.143***	0.050	0.019	−0.021	0.030	**3%**
Alcohol use	0.044	−0.186***	−0.108***	0.050	0.093***	0.064**	0.041	**5%**
Drunkenness	0.036	−0.168***	−0.090***	0.033	0.077**	0.037	0.046	**4%**
Cannabis use	0.017	−0.130***	0.015	0.076**	0.038	−0.035	0.106***	**2%**

*p < 0.05; **p < 0.01; ***p < 0.001. Statistically significant correlations are boldfaced.

## Discussion

The present study assessed the associations between EI and the frequency of substance use on a representative sample of Hungarian adolescents. According to the correlation analyses, the level of EI showed a weak relationship with substance use habits. While a higher level of emotional regulation skills related to lower frequency of substance use, a higher level on the adaptability scale (which relates to better problem-solving skills) is associated with more frequent substance use. Although not in every case, better interpersonal competencies (which relate to a higher level of empathy and helping behavior) associated with less frequent substance use. These findings were also supported by SEM and multiple linear regression analysis. Although only at a low level, stress management, interpersonal EQ, and adaptability scales were significant predictors of legal and illegal substance use habits. More difficulty in emotion regulation and empathy predicted a higher frequency of tobacco use, alcohol use, cannabis use, drunkenness, and binge drinking. However, EI explained only a small amount of the variance of substance use habits. These weak relationships are in line with the results of earlier studies, both on alcohol and tobacco use (51, 52). However, all of the factors of the Multifactor Emotional Intelligence Scale (70) had a negative association with alcohol use. EQ-i YV(S) factors have more differentiated associations with the frequency of drinking alcohol. Based on these results, it is concluded that only specific emotional and social competencies (such as emotion regulation and empathy) are risk factors of alcohol use. These results are in line with studies concerning emotion regulation strategies of adolescents and their alcohol use habits. Woods-Jaeger et al. (71) found that a limited access to emotion regulation strategies was a risk factor for more prevalent alcohol use. Furthermore, a very recent study (72) highlighted that more adaptive emotion regulation strategies (such as ‘reappraisal’) correlated with a lower frequency of alcohol use. Therefore, alcohol prevention programs should focus more on these specific types of emotion regulation skills (73).

The originality of the present study was the exploration of the relationship between adolescent *illicit drug* use and EI. As expected, a lower level of emotional regulation was associated with a higher frequency of drug use. Contrary to expectations, better problem-solving and adaptability predicted more frequent drug use. The former result can be explained by self-medication theory (31), because problems in stress management can lead to more frequent drug use. Therefore, it is proposed that the adaptability scale refers to adolescent problem-solving, creativity, and openness to new solutions (e.g., “I can easily use different ways of solving problems,” or “when answering hard questions, I try to think of many solutions”). These are in line with earlier results concerning openness and sensation-seeking since both of these traits are associated with substance use (74, 75). It is well documented that adolescents are characterized by high levels of openness (76, 77) and experimenting with psychoactive substances is also a normal part of this developmental period. The level of adaptability and a higher frequency of drug use is similar to the concept of ‘*ego-resilience*’ (78). Highly ego-resilient individuals are characteristically able to modify their level of control, either up or down, according to the situational context. They can strictly control themselves in situations where it is necessary, but in other cases where it is more adaptive, they can easily lose control. It means that they are not always overcontrolled (as inhibited, anxious, obsessive) and not always undercontrolled (as impulsive, disorganized, nonconforming). Shedlar and Block (79) defined and followed this up this proposition by carrying out a longitudinal study with three groups of adolescents according to their cannabis use habits: abstainers, experimenters, and frequent users. They found that the experimenters (rather than the abstainers) adapted the most to the challenges and problems of adult life. The abstainers who never experimented with drugs were more anxious and lacking in social skills. The present authors assume that those students who had a higher score on the adaptability scale may be more ego-resilient because they are more open and flexible in different situations. If this relationship is accepted, then it is understandable why these adolescents had more frequent drug use. On the other hand, the adaptability scale (as openness to new experiences and solutions) can be related also to nonconformity which was earlier found as a risk factor to substance use (80, 81). These associations more deeply explain the positive relationship between a higher frequency of drug use and better adaptability skills.

Another novelty of the present study was testing the individual effects of EI on substance use habits above other psychological factors such as self-esteem and depressive symptomatology. Findings indicated that depressive tendencies only predicted past month prevalence of cannabis use (i.e., the higher the level of depressive symptomatology, the higher the frequency of cannabis use). These results are similar to previous research findings (82–84). Self-esteem only predicted alcohol use habits (but only a positive relationship—higher levels of self-esteem predicted more frequent alcohol use and drunkenness). This finding is contrary to other studies, where low self-esteem has been found to be a risk factor for more frequent or problematic alcohol consumption [e.g., Refs. (85, 86)]. Symptoms of depression and self-esteem explained only a very small amount of the variance of substance use and their explanatory power was never higher than the power of the EI scales. Therefore, EI is not a key factor underlying substance use habits, but it has an individual effect beyond depressive tendencies and self-esteem. These results can be applied to both drug prevention programs and interventions in substance abuse treatment. Increasing adolescents’ self-esteem and self-efficacy can be important elements of drug prevention programs (87) and interventions targeting personality risk factors such as depressive symptomatology can prevent adolescent alcohol misuse (88). At the same time, the findings of the present study show that the developing of emotional and social competencies should also be a focus on prevention programs. Improving the level of EI might increase positive affects, satisfaction with life (89, 90), and well-being (91). Furthermore, these adaptive changes can also be protective factors against substance abuse. In future studies, it would be worth examining these indirect effects of EI on substance use habits.

Our latent profile analyses supported the differentiation of emotional and social competencies underlying substance use habits. According to the frequency of alcohol use, tranquilizer use, amphetamine use, and GHB use, different patterns of emotional and social competencies were found among adolescents. Those students who had the highest risk for frequent alcohol use and tranquilizer use had more difficulties in their emotional and social abilities than average students. On the other hand, the adolescents who had very good emotional and social competencies but had difficulties in emotion regulation showed the highest prevalences of amphetamine use and GHB use. This suggests the validity of self-medication hypothesis (31), namely different emotional patterns and problems underlying different type of psychoactive substance use. In line with the results, using depressants (alcohol and tranquilizers) was more frequent among adolescents characterized by deficits in expressing and recognizing emotions. On the other hand, using stimulants was more related to difficulties of emotion regulation. The former result is supported by previous studies, where alexithymia has been found to have a positive relationship with regular and problematic alcohol use (92). These results also emphasize the important role of emotional improvement in drug prevention programs. Developing these emotional abilities can be a protective factor against early onset of substance use and later drug abuse. As the National Institute on Drug Abuse (93) recommends, drug prevention programs should focus on improving social-emotional learning, emotional awareness, and social problem-solving too. According to the results of this study, helping children to be more effective in different emotional competencies may support them in resisting the use of different types of substance more effectively.

A few limitations also need to be considered. First, adolescents were asked about their substance use habits including illicit drug use. Although students were informed about the anonymity of their participation, some students may still have reported socially desirable responses. The self-report nature of the data also means that other well-known biases that may have occurred (e.g., memory recall biases). Second, the EQ–i YV (S) is a self-rated questionnaire, and there is always the possibility that respondents may not have perceived their emotional competencies adequately or may have tried to appear more competent than they are. Furthermore, as Grubb and McDaniel (94) emphasized, this method leaves a possibility for cheating or striving to form a desirable impression. Third, our study was cross-sectional, so any cause-and-effect relationships cannot be determined. Longitudinal studies are needed to demonstrate any causality between the variables examined in the present study. Although gender and age were controlled for in the regression models, some further moderator variables need to be considered in the future. For instance, the chronotype of adolescents moderates the relationships between EI and substance use because morningness and eveningness are important factors underlying emotional processes, ability and trait EI, and substance use (95–98). Finally, although the sample was nationally representative, it may not be representative of non-Hungarian adolescents so further research on other cultures and nationalities is needed. Despite these limitations, the present study has many strengths (including the sample size and representativeness of the data) and demonstrates that EI is not a key factor underlying substance use habits, but has an individual effect on substance use beyond depressive tendencies and self-esteem.

## Ethics Statement

This study was carried out in accordance with the recommendations of the guidelines by the Research Ethics Committee of the Faculty of Education and Psychology of the Eotvos Lorand University and the Declaration of Helsinki with written informed consent from all subjects. All subjects gave written informed consent in accordance with the Declaration of Helsinki. The protocol was approved by the Research Ethics Committee of the Faculty of Education and Psychology of the Eotvos Lorand University.

## Author Contributions

BK developed the theoretical model and research planning and wrote the preliminary versions of the paper. BP participated in research planning, and she conducted the data collection. BK and RU participated in data analyses and data interpretation. ZD supervised the entire research project and the final version of the paper. MR participated in language-editing. MG edited and supervised the final version of the paper.

## Funding

BK acknowledges the financial support of the János Bolyai Research Fellowship awarded by the Hungarian Academy of Sciences. The study was also supported by the Hungarian National Research, Development and Innovation Office (grant numbers: K111938, KKP126835). The study was completed with the support of the Hungarian Ministry of Human Capacities (ELTE Institutional Excellence Program, 783-3/2018/FEKUTSRAT).

## Conflict of Interest Statement

The authors declare that the research was conducted in the absence of any commercial or financial relationships that could be construed as a potential conflict of interest.
